# Skp2 inhibitor SKPin C1 decreased viability and proliferation of multiple myeloma cells and induced apoptosis

**DOI:** 10.1590/1414-431X20198412

**Published:** 2019-04-25

**Authors:** Ying Yang, Wei Yan, Zhuogang Liu, Minjie Wei

**Affiliations:** 1 Department of Hematology, Shengjing Hospital of China Medical University, Shenyang, Liaoning, China; 2 Department of Pharmacology, School of Pharmacy, China Medical University, Shenyang, Liaoning, China

**Keywords:** Skp2, SKPin C1, Multiple myeloma, p27, Ubiquitination

## Abstract

Multiple myeloma (MM) is a malignant neoplasm of plasma, and exhibits several harmful effects including osteolytic injuries, hypercalcemia, and immune dysfunction. Many patients with MM succumb to the underlying malignancy. An S-phase kinase-related protein 2 (Skp2) inhibitor, designated SKPin C1, has been developed and confirmed to have an inhibitory effect on metastatic melanoma cells. This study aimed to determine the effect of SKPin C1 on MM. Normal B lymphocytes, THP-1 cells, and MM U266 and RPMI 8226 cells were exposed to various dosages of SKPin C1 for 48 h. Cell proliferation was determined by MTT, EdU staining, and cell cycle assays. Western blot assays were performed to assess intracellular protein levels of Skp2, p27, and cleaved caspase-3. The amount of ubiquitin attached to p27 was determined using an immunoprecipitation assay. The viability of U266 and RPMI 8226 cells was significantly inhibited by 10 μM SKPin C1 and the inhibitory effect was enhanced with increasing doses of SKPin C1. In contrast, 50 μM SKPin C1 only marginally decreased viability of normal B lymphocytes in 12 h. Skp2 and p27 expression in U266 and RPMI 8226 cells was higher and lower, respectively, than that in the normal B lymphocytes. Treatment with SKPin C1 or Skp2 knockdown increased p27 protein levels in U266 and RPMI 8226 cells by preventing p27 from being ubiquitinated, which slowed the cell cycle, inhibited cell proliferation, and triggered apoptosis. Therefore, this study suggested SKPin C1 as a potent inhibitor against aberrant proliferation and immortalization of MM.

## Introduction

Multiple myeloma (MM) is a malignant neoplasm of plasma cells derived from a single clonal expansion in the bone marrow (1). The median age in patients with MM at the time of diagnosis is 65 years. MM accounts for 10% of all hematological cancers and 1% of all cancers (2,3). The annual incidence of MM is 7.74 per 100,000 individuals, while approximately half of the patients (3.52 per 100,000 individuals) die due to MM impairment (2,3). MM is often associated with multiple osteolytic injuries, hypercalcaemia, renal failure, and anemia (1). In addition, patients with MM easily develop immunodeficiency and various bacterial infections because the production of normal B lymphocytes is inhibited. Transplantation of hematopoietic stem cells has become the preferred treatment, but the medical expenses are very high. To date, introduction of chemotherapy remains the major treatment strategy for MM. However, many patients show refractory/relapsed MM after chemotherapy for a finite period of time.

S-phase kinase-related protein 2 (Skp2) is a key cell cycle regulator, which recently has been suggested as an important therapeutic target for cancers (reviewed in (4)). Skp2, a component of the SCFSkp2 ubiquitin E3 ligase complex, is responsible for recruiting substrate proteins for their ubiquitination and subsequent degradation by the 26S proteasome. As Skp2 mediates the ubiquitination degradation of many negative regulators of cell proliferation such as p27, p21, p53, and p57 (also known as CDKN1C), upregulated Skp2 is associated with rapid growth and immortalization of cancer cells (5). Malek et al. (6) recently reported that high expression of Skp2 in CD138+ cells of MM patients is correlated with decreased progression-free and overall survival. In addition, it has been identified that CDC28 kinase subunit 1 (CKS1B) is also inversely associated with survival in newly diagnosed MM, and high nuclear expression of CKS1B is an adverse prognostic factor for relapsed/refractory MM patients (7,8). CKS1B is a necessary cofactor of cullin-RING ubiquitin E3 ligases, which participate in Skp2-mediated ubiquitination, especially that of p27; therefore, CKS1B also regulates cellular entry into S phase and possesses anti-apoptotic activity (7,8). All the above-mentioned evidence suggests that Skp2-mediated p27 ubiquitination is an important mechanism underlying uncontrolled proliferation and immortalization.

Previous studies have developed a small molecule inhibitor of Skp2, named SKPin C1 (9). This Skp2 inhibitor protects p27 and p21 from ubiquitination degradation, thereby decreasing the viability of metastatic melanoma cell lines 501 Mel, SK-MEL-147, and SK-MEL-173 and inducing their apoptosis (9). The present study aimed to determine the inhibitory effect of SKPin C1 on the proliferation of MM U266 and RPMI 8226 cell lines, and provides important data to support the promising effect of SKPin C1 as a drug in the treatment of MM.

## Material and Methods

### Cell culture

Normal B lymphocytes were isolated from six healthy participants including three males and three females. All samples were obtained after receiving written informed consent from participants. Peripheral blood mononuclear cells (PBMCs) were isolated by centrifuging 20 mL of fresh blood overlaid with the same volume of 100% Ficoll (GE Healthcare, China) at 400 *g* for 40 min at 37^o^C. Peripheral B cells were isolated from PBMCs using MACS isolation kit (Miltenyi Biotec, China), according to the manufacturer's instructions. Consequently, approximately 3.0×10^6^ of B lymphocytes were isolated from 1×10^8^ of PBMCs.

Multiple myeloma U266 and RPMI 8226 cells as well as human peripheral blood mononuclear cell line THP-1 were purchased from the American Type Culture Collection (USA). All cells were cultured in Roswell Park Memorial Institute (RPMI)-1640 (Sigma, USA) containing 10% fetal bovine serum (Invitrogen, USA) and 1% penicillin/streptomycin (Life Technologies, USA) at 37°C under a humidified atmosphere of 5% CO_2_. The medium was changed every 2 to 3 days during the cell culture.

SKPin C1 was purchased from Selleck Company (No. S8652, China). B lymphocytes, U266, RPMI 8226, and THP-1 cells were exposed to various dosages of SKPin C1 (0, 5, 10, 25, and 50 μM) for 48 h. Thereafter, their viability was measured using (4,5-dimethylthiazole-yl)-2,5-diphenyl tetrazolium bromide dye (MTT) (Sigma-Aldrich, USA) according to a standard protocol. The absorbance at 490 nm was measured by a microplate reader (Bio-Rad, USA). The protein levels of Skp2 and p27 in cells were assessed by western blotting. The following assay was performed at 12 h after the SKPin C1 treatment.

### Western blot assay

The cells were lysed and boiled at 96°C for 5 min, before loading onto four 20% SDS-polyacrylamide gels. Proteins were separated by electrophoresis in a Mini-PROTEAN Tetra cell chamber and transferred to polyvinylidene difluoride membranes. Then, the membranes were blocked in 5% non-fat milk (Yili Milk Company, China) in Tris-buffered saline-Tween (TBS-T) for 1 h at room temperature, and incubated with primary antibodies against Skp2 (ab68455, Abcam, UK), p27 (ab215434, Abcam), caspase-3 (ab2302, Abcam), and β-actin (ab8227, Abcam) overnight at 4°C with gentle shaking. Secondary antibodies conjugated to horseradish peroxidase (HRP) were applied for 1 h at room temperature, and immunoreactive bands were developed using enhanced chemiluminescence (Thermo Fisher Scientific, USA). The obtained bands were quantified in ImageJ x64 by normalizing to loading control and calculating band density relative to untreated control. Resulting graphs show an average of three independent donors.

### Gene silencing

U266 and RPMI 8226 cells were seeded in 12-well plates (1×10^5^ cells/well). An siRNA (5 nM) targeting Skp2 was synthesized by GenePharma Company (China) and added to cells with Lipofectamine 3000 (Invitrogen). Control cells were treated with Scramble siRNA and Lipofectamine 3000. After 8 h, the cells were collected for cell viability, cell cycle, EdU staining, and immunoprecipitation assays.

### Flow cytometry analysis

For cell cycle analysis, cells were harvested and fixed with 70% cold ethanol at 4°C overnight. After being washed in PBS, the cells were incubated in 1 mL of staining solution (20 mg/mL propidium iodide; 10 U/mL RNaseA) (Sigma) at room temperature for 30 min. For apoptosis analysis, cells were stained using Annexin V-FITC/PI Apoptosis Detection Kit I (Kaiji Biological Inc., China) according to the manufacturer's instructions. Then, the samples were measured by FACS Calibur flow cytometry (BD, USA), and then analyzed by the software FlowJo V10 (FlowJo, LLC, USA).

### EdU staining assay

Cells were treated with EdU for 2 h, washed with 3% BSA three times, and fixed with 4% paraformaldehyde for 10 min. After washing with 3% BSA three times, cells were permeabilized with 0.4% Triton X-100 for 15 min. Cells were then incubated with EdU staining cocktail kept from light at room temperature for 30 min. After washing with 3% BSA, samples were counterstained with 1× Hoechst 33342 for 10 min. Images were acquired by fluorescence microscope (Olympus BX61, Japan). Fluorescence of cells was measured using the flow cytometer.

### Immunoprecipitation assay

Cells were lysed with immunoprecipitation assay lysis buffer (RIPA, Sigma). Cell lysates with equal amounts of protein (500 μg) were then incubated with nickel beads conjugated to anti-p27 antibody (Abcam) for 3 h, followed by washing with IP buffer (50 mM Tris, pH 7.5, 5 mM EDTA, 150 mM NaCl, 0.5% NP-40). Bound proteins were detected by western blotting using anti-ubiquitin primary antibody and HRP-conjugated secondary antibody.

### Statistical analysis

Statistical analysis was performed using the SPSS statistical software package (version 16.0, USA). Data are reported as means±SD and were evaluated by one-way analysis of the variance (ANOVA) followed by Dunnett's *post hoc* test. Every experiment was repeated at least three times. P<0.05 was considered to be statistically significant.

## Results

### Effect of SKPin C1 on cell proliferation

**Figure 1.**
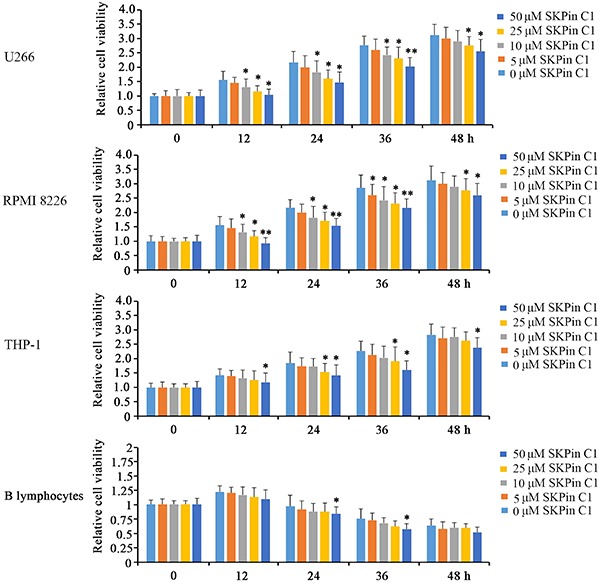
Effect of different dosages of SKPin C1 on the viability of B lymphocytes, U266, RPMI 8226, and THP-1 cells. Cell viability was measured by MTT at different time points. Data are reported as means±SD. *P<0.05, **P<0.01 *vs* non-treated group (control, 0 μM) (ANOVA).

This study initially evaluated the toxic effect of SKPin C1 on B lymphocytes, U266, RPMI 8226, and THP-1 cells. These cells were subjected to different dosages of SKPin C1. As indicated by cell the viability test, treatment with 10 μM SKPin C1 for 12 h significantly decreased the viability of U266 and RPMI 8226 cells (P<0.05, Figure 1), and the inhibition was dose-dependently increased by SKPin C1. Although U266 and RPMI 8226 cells were sensitive to SKPin C1, B lymphocytes and THP-1 showed a high tolerance, because treatment with SKPin C1 at the tested range of concentrations for 12 h did not significantly affect B lymphocyte viability, and only the highest dose of SKPin C1 (50 μM) decreased THP-1 cell viability (P<0.05). After incubation for 48 h, 10 μM SKPin C1 failed to decrease U266 and RPMI 8226 cell viability, but 25 and 50 μM SKPin C1 was still effective in the inhibition of their cell viability (P<0.05). THP-1 cells also showed decreased cell viability after treatment with 25 and 50 μM SKPin C1 for 24 and 36 h (P<0.05). Since the highest dose of SKPin C1 (50 μM) exerted a toxic effect on B lymphocytes following the treatment for 24 and 36 h (P<0.05), SKPin C1 at a dose of 25 µM was used in the majority of functional assays in B lymphocytes, U266, and RPMI 8226 cells.

**Figure 2.**
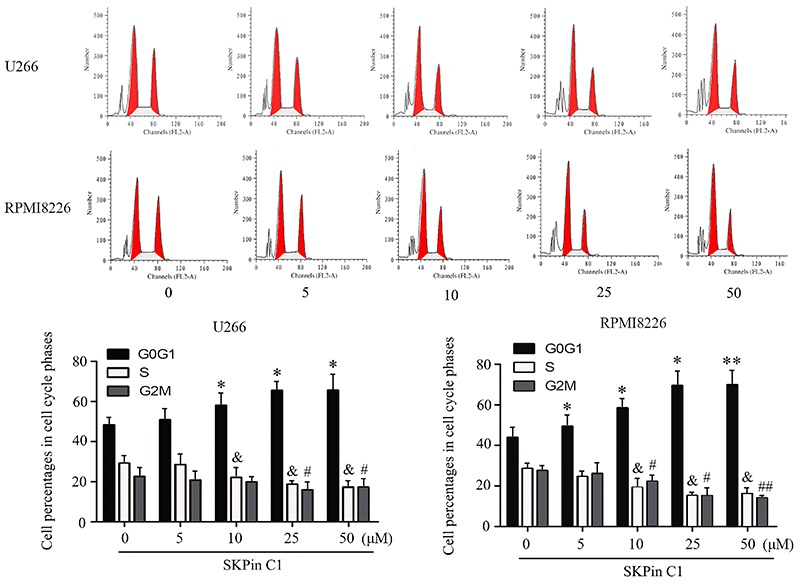
SKPin C1 retarded the cell cycle of U266 and RPMI 8226 cells. B lymphocytes, U266, and RPMI 8226 cells were subjected to different dosages of SKPin C1 for 12 h. The cell cycle was evaluated using flow cytometry analysis. Data are reported as means±SD. *P<0.05, **P<0.01, ^#^P<0.05, ^##^P<0.01, ^&^P<0.05 *vs* non-treatment group (control, 0 μM) (ANOVA).

The present study additionally tested the effect of SKPin C1 on U266 and RPMI 8226 cell cycle. With the increased dosages of SKPin C1, the percentages of U266 and RPMI 8226 cells in the G0/G1 phase were also increased (P<0.05 or P<0.01, Figure 2), while the percentages in S and G2/M phases were decreased (P<0.05 or P<0.01). These results suggested that SKPin C1 retarded U266 and RPMI 8226 cell cycle.

### Expression profiles of SKP2 and p27

**Figure 3.**
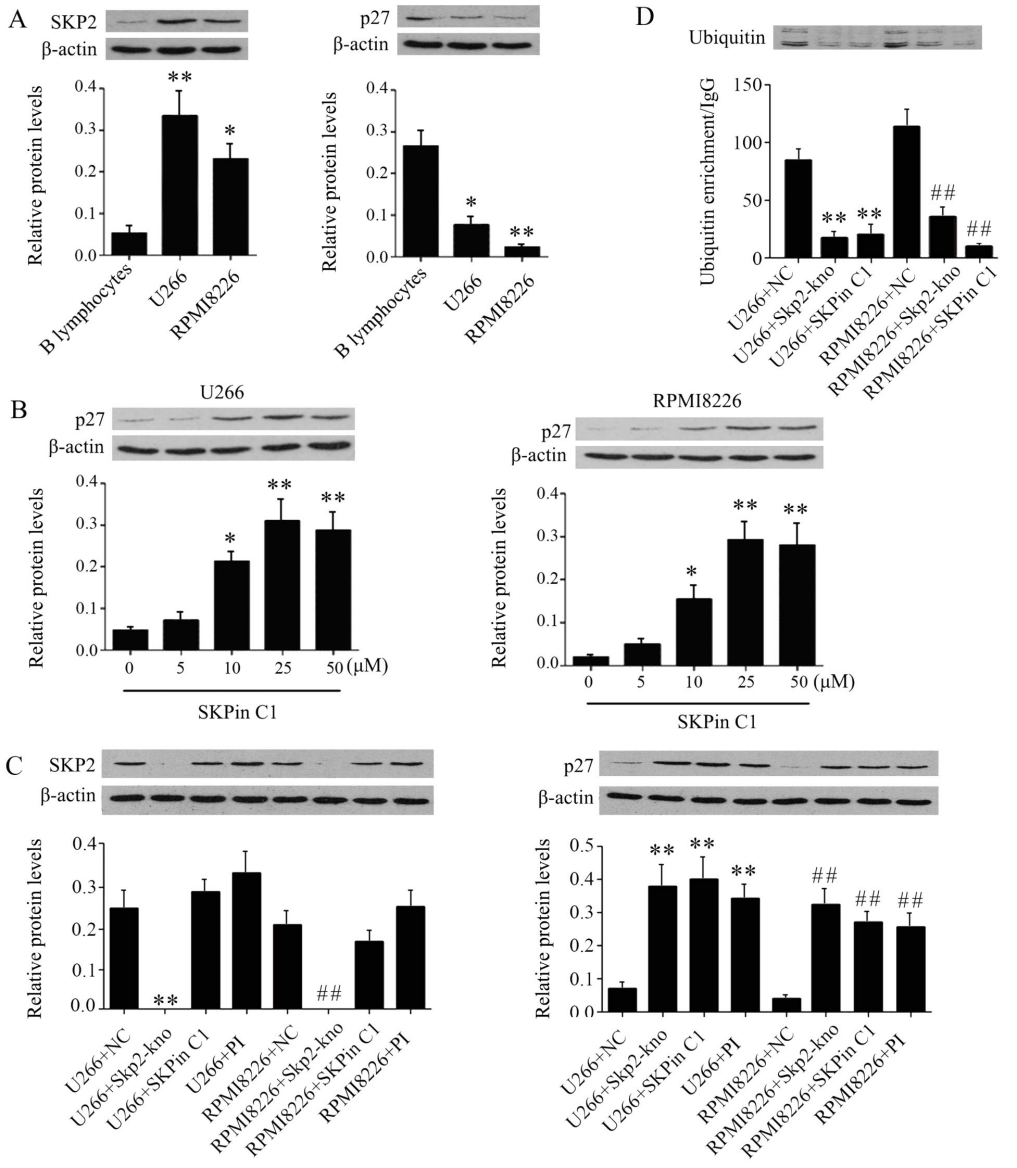
SKPin C1 increased p27 protein levels in U266 and RPMI 8226 cells by inhibiting ubiquitination, evaluated by western blot assay. **A**, Protein levels of SKP2 and p27 in U266 and RPMI 8226. **B**, Intracellular p27 protein levels in U266 and RPMI 8226 cells exposed to different dosages of SKPin C1. **C**, Protein levels of SKP2 and p27 in U266 and RPMI 8226 cells treated with 25 μM of SKPin C1 or 25 μM of bortezomib, or transfected with the siRNA that targeted SKP2. **D**, U266 and RPMI 8226 cells were treated with 25μM of SKPin C1, or transfected with the siRNA targeted SKP2. The amount of ubiquitin protein combined to p27 was determined using an immunoprecipitation assay. Data are reported as means±SD. *P<0.05 and **P<0.01 *vs* control group of U266 cells; ^##^P<0.01 *vs* control group of RPMI 8226 cells (ANOVA). SKP2-kno: SKP2 knockdown; PI: proteasome inhibitor, namely bortezomib.

The sensitivity of B lymphocytes, U266, and RPMI 8226 cells to SKPin C1 is probably associated with the different expression levels of SKP2 and p27 in these cells. In accordance with this hypothesis, western blot analysis showed that SKP2 was highly expressed (P<0.05 or P<0.01, Figure 3A), but p27 was lowly expressed in U266 and RPMI 8226 cells (P<0.05 or P<0.01) compared to B lymphocytes.

### SKPin C1 increased the accumulation of p27 in cells

We added different doses of SKPin C1 to U266 and RPMI 8226 cells to further determine the effect of SKPin C1 on p27 protein level. Western blot analysis showed that p27 protein enrichment was increased by SKPin C1 in a dose-dependent manner (P<0.05 or P<0.01, Figure 3B). However, the SKP2 protein level in these cells was not significantly changed by SKPin C1 (data not shown). This study knocked-down SKP2 in U266 and RPMI 8226 cells to compare the effect of SKP2 depletion with that of SKPin C1 treatment. SKPin C1 treatment had no effect on SKP2 expression. The protein level of p27 was increased by both SKP2 knockdown and SKPin C1 treatment (P<0.01, Figure 3C). Bortezomib is a proteasome inhibitor. It was supposed to increase p27 protein level in U266 and RPMI 8226 cells by the inhibition of p27 ubiquitination. Treatment with Bortezomib (25 µM) only moderately increased the SKP2 protein level, but significantly elevated p27 protein level (P<0.01). Results from immunoprecipitation assay revealed that the amount of ubiquitin protein combined to p27 was decreased with SKPin C1 treatment (P<0.01, Figure 3D). Consistent with the SKPin C1 treatment, SKP2 knockdown also reduced the accumulation of ubiquitin protein at p27.

### SKPin C1 modulated cell proliferation and apoptosis

**Figure 4.**
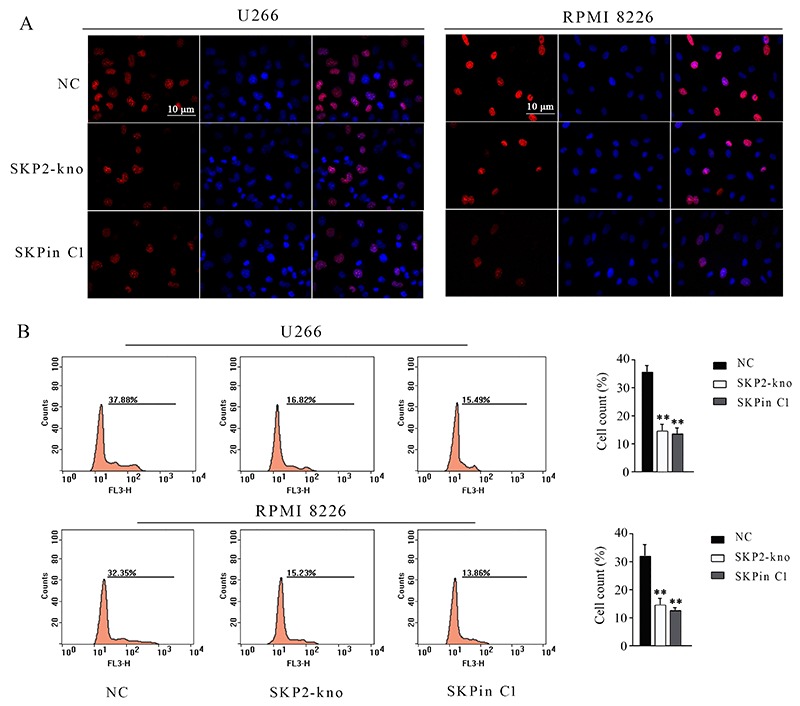
Effect of SKPin C1 treatment and SKP2 depletion on the proliferation of U266 and RPMI 8226 cells treated with 25μM SKPin C1 or transfected with the siRNA that targeted SKP2, evaluated by Edu staining assay (**A**). Flow cytometry analysis of the number of cells with positive Edu staining (**B**). Data are reported as means±SD. **P<0.01 *vs* control group (NC) (ANOVA). SKP2-kno: SKP2 knockdown.

**Figure 5.**
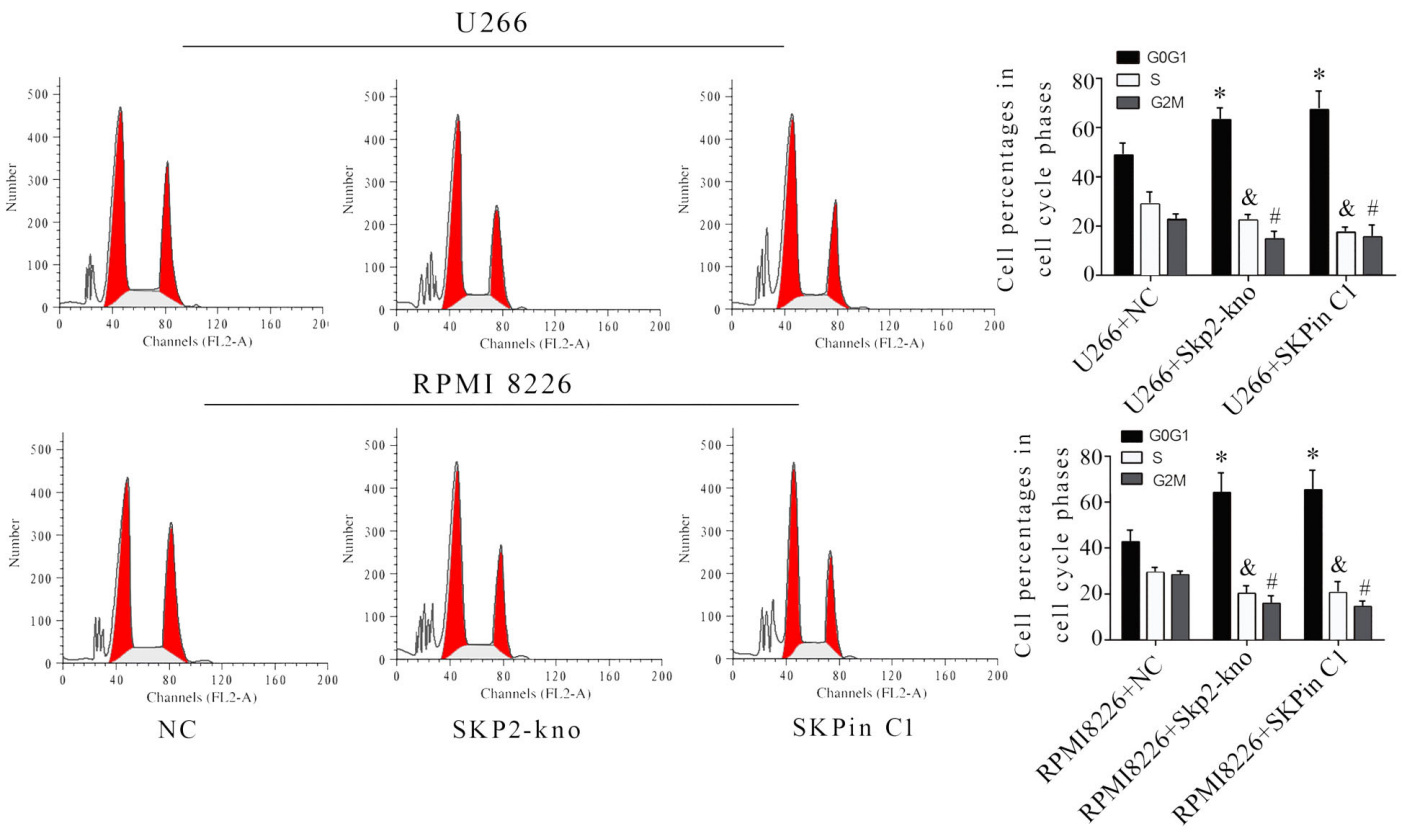
Effect of SKPin C1 treatment and SKP2 depletion on the cell cycle of U266 and RPMI 8226 cells treated with 25μM SKPin C1 or transfected with the siRNA that targeted SKP2, evaluated using flow cytometry analysis. Data are reported as means±SD. *P<0.05, ^#^P<0.05, ^&^P<0.05, *vs* control (NC) (ANOVA). SKP2-kno: SKP2 knockdown.

In addition to cell viability testing, we also performed an EdU staining assay to evaluate the inhibitory effect of SKPin C1 on U266 and RPMI 8226 cell proliferation. It was observed that SKPin C1 inhibited EdU staining of U266 and RPMI 8226 cells, having a similar effect to SKP2 knockdown (Figure 4A). Flow cytometry analysis also indicated that SKPin C1 treatment and SKP2 knockdown decreased the number of positive cells with EdU staining (P<0.01, Figure 4B). Analysis of the cell cycle showed that both SKPin C1 treatment and SKP2 knockdown increased the percentages of cells at G1 phase but decreased the cell percentages at S and G2/M phases (P<0.05, Figure 5).

**Figure 6.**
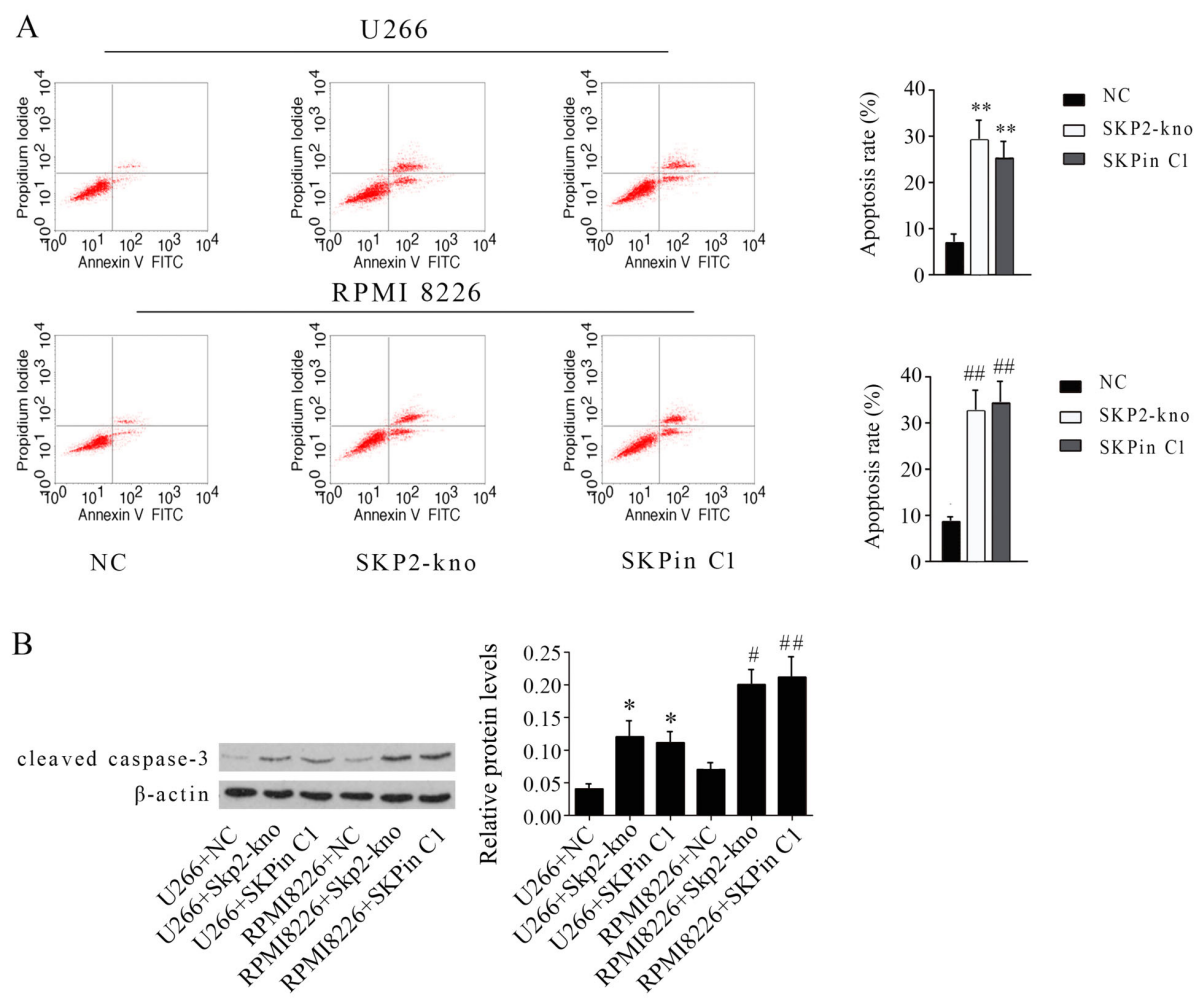
Effect of SKPin C1 treatment and SKP2 depletion on the apoptosis of U266 and RPMI 8226 cells treated with 25 μM SKPin C1 or transfected with the siRNA that targeted SKP2, evaluated using flow cytometry assay (**A**). Protein levels of cleaved caspase-3 were evaluated using western blot measurement (**B**). Data are reported as means±SD. *P<0.05, **P<0.01 *vs* control group of U266 cells; ^#^P<0.05, ^##^P<0.01 *vs* control group of RPMI 8226 cells (ANOVA). SKP2-kno: SKP2 knockdown.

As assessed by flow cytometry assay, apoptosis rates of U266 and RPMI 8226 cells were notably increased by SKP2 knockdown and SKPin C1 treatment (P<0.05, Figure 6A). In addition, this study measured the protein level of cleaved caspase-3 to evaluate apoptosis rate. As with SKP2 knockdown, SKPin C1 treatment increased protein levels of cleaved caspase-3 in U266 and RPMI 8226 cells (P<0.05 or P<0.01, Figure 6B).

## Discussion

Tumor development is characterized by aberrant proliferation and immortalization of tumor cells due to disordered regulation of the cell cycle. Skp2 is a key regulator of the cell cycle, because many molecules downstream of Skp2 function as important modulators in most stages of the cell cycle (4). Skp2 has been found to be up-regulated in many types of tumors including MM, and up-regulated Skp2 is associated with rapid tumor progression and short survival time of patients (6). A small molecule inhibitor of Skp2, named SKPin C1, was developed and confirmed to demonstrate an inhibitory effect on cell proliferation in human skin malignant melanoma cells. The present study tested the effect of SKPin C1 on normal B lymphocytes and MM cell lines. Results showed that a relatively low concentration of SKPin C1 significantly inhibited viability of U266 and RPMI 8226 cells, but a relatively high concentration of SKPin C1 only marginally attenuated the viability of normal B lymphocytes. These data suggested that U266 and RPMI 8226 cells were more sensitive to SKPin C1 than normal B lymphocytes.

Interestingly, SKPin C1 had no effect on the protein levels of Skp2 but remarkably increased p27 protein levels. These data are consistent with the results of a study performed in human skin malignant melanoma cells. Wu et al. have reported that SKPin C1 interfered with the interaction of Skp2 with p27, thereby preventing Skp2-mediated p27 ubiquitination and degradation (9). To determine that SKPin C1 increase of p27 in MM cells is also associated with the blockage of p27 ubiquitination, we performed an immunoprecipitation assay and measured the amount of the ubiquitinated protein combined to p27. Results indicated that SKPin C1 indeed prevented p27 ubiquitination in the MM cells. p27 functions as a negative regulator of the cell cycle, thus it is regarded as a tumor suppressor gene. p27 can regulate the activity of CDKs that inhibit the activity of cyclin E and cyclin A, thereby regulating the metastasis of G1 and S phases. In the present results, the percentages of U266 and RPMI 8226 cells at G1 phase were notably increased by SKPin C1. As a result, the percentages of U266 and RPMI 8226 cells at S and G2/M phases were correspondingly decreased by SKPin C1. These data suggest that SKPin C1 inhibited the proliferation of U266 and RPMI 8226 cells by arresting the cell cycle. We found that Skp2 was highly expressed but p27 was lowly expressed in U266 and RPMI 8226 cells compared to those in normal B lymphocytes. This is likely an important reason that U266 and RPMI 8226 cells are more sensitive to SKPin C1 than normal B lymphocytes.

Previous studies have used various inhibitors of Skp2 to retard the growth of tumor cells or induce apoptosis. Oh et al. (10) recently found an inhibitor that binds to Skp2 and interferes with the Skp2/p300 interaction through an affinity-based high-throughput screen of a combinatorial cyclic peptoid library. This inhibitor has been confirmed to promote p300-mediated p53 acetylation and consequent p53-mediated apoptosis in cancer cells, although it has no effect on Skp2 proteolytic activity. Huang et al. (11) recently added an inhibitor of Skp2, named C25, to human prolactinoma cells, which sensitized bromocriptine-induced apoptosis by downregulating Skp2 and increasing the accumulation of Bax, cytochrome C, and cleaved caspase-3 in cells. Malek et al. (6) identified a novel compound, designated DT204, through chemical library screens. DT204 reduced Skp2 binding to Cullin-1 and Commd1, and synergistically enhanced bortezomib-induced apoptosis in MM cells. Bortezomib is a proteasome inhibitor that has been approved by the Food and Drug Administration for treatment of patients with MM. Although bortezomib is not a specific inhibitor of Skp2, it protected p27 from Skp2-mediated ubiquitination by inhibiting the proteasome function, resulting in suppressed colony formation ability of chronic myelogenous leukemia cells and increased apoptosis rate (12). This study compared the effects of bortezomib and SKPin C1 on the regulation of Skp2 and p27. Similar to SKPin C1, bortezomib had no significant effect on the protein level of Skp2 in U266 and RPMI 8226 cells. Both SKPin C1 and bortezomib increased p27 protein levels with the more profound effect observed in SKPin C1.

In the present study, SKPin C1 not only retarded the proliferation of MM cells, but also stimulated caspase-3-mediated apoptosis. We speculate that the up-regulation of p27 was the key mechanism for SKPin C1 triggering apoptosis, because SKPin C1-mediated protection of p27 from ubiquitination and degradation exerted similar promoting effects on MM apoptosis with Skp2 knockdown. p27 has been involved in the regulation of apoptosis, in addition to the role in the regulation of cell cycle and proliferation. CpdA is also an inhibitor of Skp2, which can prevent the incorporation of Skp2 into the SCF(Skp2) ligase. CpdA inducing G(1)/S cell-cycle arrest as well as the death of MM cells is p27-dependent, but this programmed cell death was caspase-independent, and instead occurred through activation of autophagy (13). It has been reported that cell cycle arrested in late G1 or S phase can accelerate apoptosis (14,15). Many cell cycle regulators, such as CDK4/6-cyclin D and CDK2/cyclin E or A, have been confirmed to impact apoptosis (16,17). Therefore, p27 induces apoptosis by regulating the activity of these CDK/cyclins molecules (18). In addition, up-regulated p27 by the transfection of the adenoviral vector caused the down-regulation of pRb, which resulted in significant apoptosis of lung cancer cells (19). In oral and oropharyngeal squamous cell carcinomas, spontaneous apoptosis in the p27-positive tumors is higher than that in p27-negative tumors (20). Further study showed that expression of p27 in patients with oral oropharyngeal squamous cell carcinomas is positively correlated with Bax expression (20). Bax is a component of the p53-mediated apoptotic response, which is located relatively downstream of the apoptotic pathway and is associated with the acceleration of apoptosis. All this evidence suggested that increasing p27 expression induced apoptosis of cancer cells.

In summary, this study found that SKPin C1 notably decreased the proliferation of U266 and RPMI 8226 cells, and induced caspase-3-mediated apoptosis, but only moderately attenuated viability of normal B lymphocytes, suggesting a highly specific effect on MM cells. The inhibitory effect of SKPin C1 on cell proliferation is associated with its protection of p27 from Skp2-induced ubiquitination and degradation.
